# Abdominal wall reconstruction for desmoid tumour surgery: Case report

**DOI:** 10.1016/j.ijscr.2019.09.010

**Published:** 2019-09-20

**Authors:** Francesca Ascari, Silvia Segattini, Michele Varoli, Massimo Beghi, Simone Muratori, Bruno Scotto, Marco Gasperoni

**Affiliations:** General Surgery Unit, “Bernardino Ramazzini” Hospital, Via Guido Molinari 2, 41012, Carpi, MO, Italy

**Keywords:** FAP, familial adenomatous polyposis, US, ultrasonography, MRI, magnetic resonance imaging, CT, computed tomography, NSAIDs, non-steroidal anti-inflammatory drugs, Desmoid tumour, Posterior component separation, Abdominal wall reconstruction, Case report

## Abstract

**Introduction:**

Desmoid tumours are rare benign neoplasms with strong tendency to local invasion and recurrence. They usually rise from muscles and aponeurosis and could be challenging for medical treatment. We here report the case of a patient requiring the excision of part of the abdominal rectus muscle and an abdominal wall reconstruction.

**Presentation of case:**

A 38 years old women referred to our institution complaining of a painful lump in the anterior abdominal wall. She underwent ultrasonography (US), magnetic resonance imaging (MRI) and a computed tomography (CT) biopsy who showed the presence of a desmoid tumour arising in the right abdominal rectus muscle. She underwent surgical excision of the mass and an abdominal wall reconstruction according to the posterior component separation technique, with a large polypropylene mesh reinforcement. The post-operative period was uneventful and at 6 months she shows no clinical nor radiological recurrence.

**Discussion:**

Desmoid tumours are rare neoplasms rising in many anatomical spaces and requiring challenging decisions. A radical resection with free margins remains the principal determinant of outcome. Non-surgical approaches and adjuvant therapy may be useful for patients with unresectable lesions.

**Conclusion:**

The optimal treatment strategy for desmoid tumours still remains unclear. A multidisciplinary approach is always the best strategy to establish the correct treatment.

## Highlights

• Desmoid tumours are rare benign neoplasms with local invasion and recurrence.
• A margin-free excision may be challenging for the surgeon.
• If necessary, abdominal wall reconstruction can be required for rectus abdominis muscle tumours.
• A multidisciplinary approach is necessary approaching this rare disease.

## 1 Introduction

Desmoid tumours are histologically benign myofibroblastic neoplasms rising from muscular aponeuroses. These lesions are also known as deep fibromatoses or aggressive fibromatoses and they have a strong tendency to invade and recur locally after resection, with an intermediate biological behaviour between benign fibrous tumours and fibrosarcomas. Desmoid tumours could be extra-abdominal (shoulder, pelvic ring, thorax, neck and limbs), abdominal, intraabdominal, multiple, multiple familial and associated with FAP or Gardner's syndrome. They represent the 0,03% of all neoplasms and 3% of all soft tissue tumours [[1], [2], [3]]. These neoplasms are rare, the highest incidence is between the ages of 24 and 40 years with a strong prevalence among fertile aged women and during pregnancy; they are uncommon during menopause (and this corroborates the estrogen-stimulated tumour growth hypothesis) [4]. The most common site is the anterior abdominal wall, with an incidence of 50% and in this case, they measure from 5 by 15 cm in diameter [[1], [2], [3]]. The differential diagnosis includes acute hematoma, fibrosarcoma, lymphoma, rhabdomyosarcoma, liposarcoma, leiomyosarcoma, neurofibroma, benign fibrous tumour and primitive neuroectodermal tumour [5]. The desmoid tumours have three stages of evolution: in the first stage they are more cellular and have fewer areas of hyalinized collagen. In the second stage, there is an increasing amount of collagen deposition in the central and peripheral areas of tumour. In the third stage there is an increase in the fibrous composition with a decrease in cellularity and water content [6]. Despite their lack of metastatic potential, local infiltration and compression in anatomic locations with restricted access to surgical resection, may lead to fatalities [[1], [2], [3]].

According to the SCARE criteria [7], we present the case of a 38-year-old woman without history of trauma or abdominal surgery and her diagnostic and therapeutic pathway.

## 2 Presentation of case

**Fig. 1:**
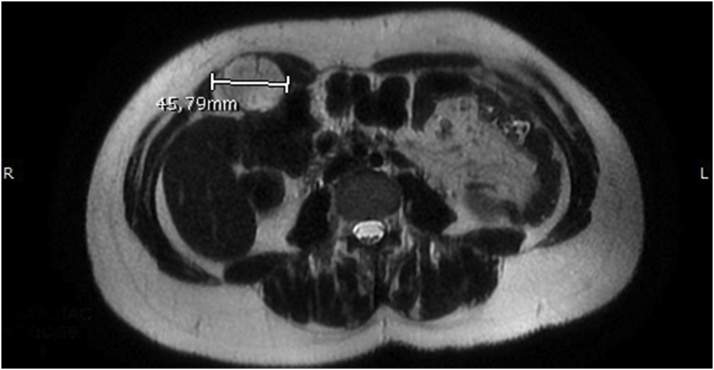
MRI detail of the desmoid tumor in the right rectus abdominis muscle.

A 38 years old woman referred to her general practitioner in August 2018 for a painful mass in the right anterolateral abdomen. On physical examination the mass was tough and fixed to the abdominal wall. In September 2018 an ultrasound examination was performed and demonstrated an oval solid mass of heterogeneous echogenicity in the context of right rectus abdominis muscles, with irregular margins and modestly vascularized (axial diameters 47 × 32 × 44 mm) [[8], [9], [10]]. In October 2018 the patient was evaluated in our Surgical Department. The mass was gradually increasing in size during that time. She had no relevant familiar history and she did not smoke, drink alcohol or take any medications. She had one child, naturally delivered and she denied any abdominal surgery or trauma. Her blood tests were within the normal range and the oncological markers resulted negative. Preoperative MRI (Fig. 1) showed an ovoid mass (35 × 45 × 46 mm) in the context of right rectus abdominis muscles. It demonstrated heterogeneous enhancement, predominantly high signal in T2-weighted images and low signal in T1-weighted images, demonstrating low vascularization [[8], [9], [10]]. In November 2018 a CT-scan guided biopsy was performed showing a desmoid tumour. In December she made a colonoscopy in order to search any sign of FAP, but it was negative.

**Fig. 2:**
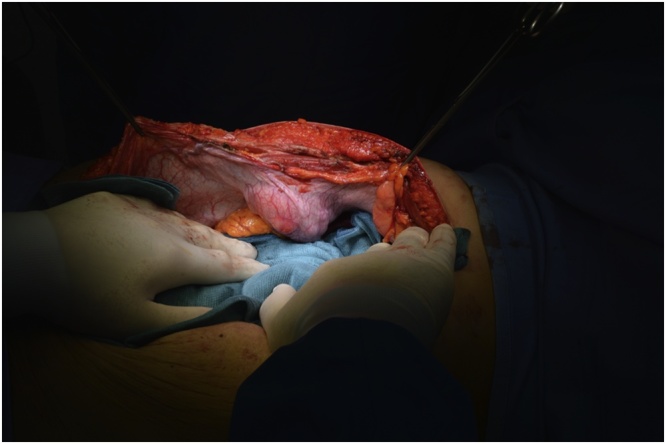
The desmoid tumor in the context of the muscle, buldging but not invading the peritoneum.

**Fig. 3:**
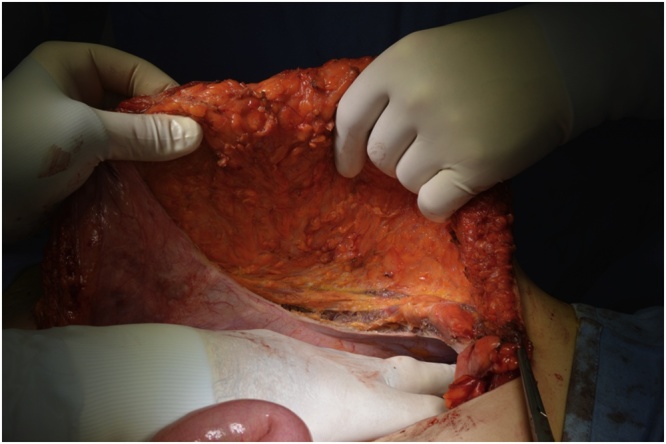
The right rectus abdominis muscle after the resection of the desmoid tumor.

**Fig. 4:**
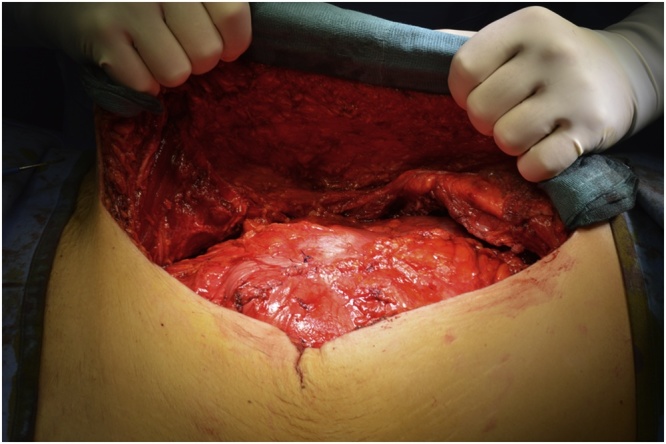
Reconstruction of the midline with posterior component separation.

**Fig. 5:**
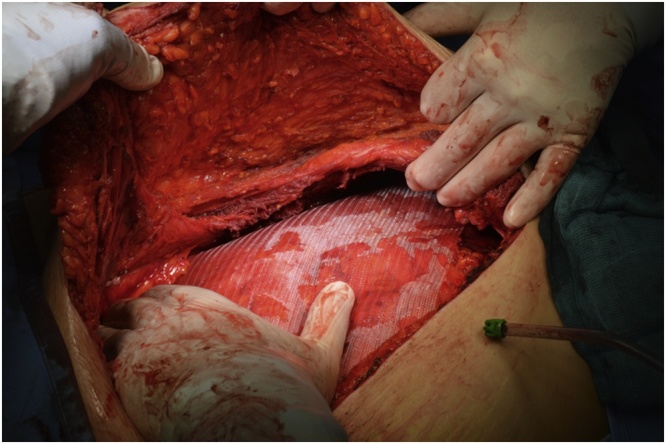
Two 20 × 30 polypropilene meshes are placed to reinforce the abdominal wall.

In January 2019 an “en-bloc” resection of the mass was performed including the peritoneum and the right rectus muscle (Fig. 2, Fig. 3), with a minimum of 2 cm margin of healthy tissue. The abdominal wall defect measured 7 × 5 cm (35 cm^2^). A posterior-component separation technique was performed, extending the dissections towards the lumbar and ileo-psoas muscles to allow the closure of the midline. A 40 × 60 cm mesh obtained suturing two 20 × 30 cm polypropylene meshes (Repol Angimesh, Angiologica, Pavia, Italy) [16,18] was placed in a retro-muscular space (Fig. 4, Fig. 5), fixed with fibrin glue (Evicel, Ethicon Johnson & Johnson, Somerville, NJ, USA) in the lumbar space and with polypropylene sutures to the Cooper ligaments and costal margins. Finally, two suction drains were placed and the anterior rectus sheet and skin were sutured.

Macroscopically the tumour was solid, with a firm texture. On the cut surface it appeared drawn, stretchy and greyish, resembling scar tissue. The histological diagnosis was of a desmoid tumour and every margin was free. The post-operative period was uneventful and the patient was discharged on the tenth post-operative day. At six months follow-up after surgery the patient is healthy, shows complete remission without any other treatment and MRI showed no local recurrence nor incisional hernias.

## 3 Discussion

Desmoid tumours are benign deep fibromatoses that originate from fascia and muscular aponeuroses, especially the rectus and internal oblique muscle and occasionally cross the midline. They have an infiltrating growth pattern and less commonly originate from the external oblique and the transversalis muscle or fascia. They are rare tumours occurring in 3.7 cases per million individuals every year. They have been correlated with female gender, FAP and occasionally with abdominal trauma [[1], [2], [3]].

Plenty of modern imaging method are used for the diagnosis. In ultrasonography desmoids have variable echogenicity, with smooth, well-defined margins. In CT scan the lesions are generally characterized by high attenuation (relative to muscle) and have either well or ill-defined margins. Even a CT scoring system has been developed, characterizing specimens according to the presence of desmoid precursor lesions (mesenteric fibrosis) and true desmoid tumours. This has provided further evidence for a stepwise progression in desmoid development. The degree of enhancement after the intravenous administration of contrast medium is variable. In MRI scans desmoid tumours have a low signal intensity in T1-weighted images (relative to muscle) and variable signal intensity on T2-weighted images (high in our case). Furthermore, MRI scans can indicate how the tumours will probably behave, with a bright signal indicating a high-water content, which has been correlated with rapid growth. Sometimes low T2 signal intensity bands are characteristic and represent foci of high concentrations of collagen deposition. However, the definitive diagnosis must be established with a histopathological analysis with diffuse cell infiltration of the adjacent tissue structures. The immunohistochemical response for actin may be partially positive and immunohistochemical muscle cell markers may distinguish desmoid tumours from fibrosarcoma [[8], [9], [10]]. In our case we strongly suspected a desmoid tumour with the MRI, but we needed to perform two CT-scan biopsies to achieve a clear histopathological diagnosis (the first report was undefined and the pathologist asked for a second biopsy); this caused a delay in the diagnosis, but after that we promptly arranged a correct therapy plan.

The local invasive nature and high rates of recurrence of this type of lesion, force surgeons to manage with difficult choices and require complicated treatments. There are several possibilities as surgical resection, radiotherapy, hormonal therapy (tamoxifen or toremifene) and use of non-steroidal anti-inflammatory drugs (as indomethacin, sulindac or celecoxib). We could say that chemotherapy is usually reserved for patients with significant symptoms that do not respond to milder approaches such as NSAIDs and tamoxifen [[1], [2], [3],[11], [12], [13], [14], [15], [16], [17], [18], [19], [20]]. Abdominal wall reconstruction may be achieved by direct repair (with sutures) or by using synthetic materials (meshes) or myocutaneous flaps when the defect is extensive. In our case we performed the resection of the upper part of the right rectus abdominis muscle including the tumour with at least 2 cm-margin of healthy tissue. The resultant abdominal wall defect was of 7 × 5 cm and we decided that a reconstruction with mesh was necessary for two reasons: first, restore the abdominal wall physiology after the resection of the rectus abdominis muscle; secondly, reinforce the abdominal wall to prevent incisional hernias. We must say that prosthetic materials are more susceptible to bacterial infection and other complication, although newly developed materials have exhibited encouraging experimental results. The prosthesis size must allow at least 3 cm overlap on the parietal defect. Post-operative complications could be muscular dysfunction with a subsequent decrease of pulmonary and respiratory function, bleeding, seroma, infections, post incisional hernia and abdominal wall bulging [2,3,13,16,18].

Radiation therapy has been used predominantly for the extra-abdominal desmoid tumours treatment and has resulted in an improvement of local control by reducing local recurrence rates. External beam irradiation or brachytherapy may be used alone, especially in patients with unresectable lesions, even if they are correlated with high failure rates. They may also be used either as new-adjuvant or adjuvant therapies following incomplete surgical resection [12,14].

The recurrence rate is 20%–77% depending on location, extent and completeness of initial resection. Abdominal wall desmoid tumours have a significantly lower recurrence rate (20–30%) and usually becomes evident within six months after excision or in connection with subsequent gestations or deliveries. Metastatic disease has not been reported with desmoid tumour [11]. In women desmoid tumours can spontaneously regress with menopause or ovariectomy [4].

Radical tumour resection with free margins has typically been recommended as the first-line therapy; however, an increasing number of surgeons are using “wait and see” strategies because an optimal management strategy has not been defined. Even the UK guidelines, referred to the recent European Consensus, stated that watchful waiting should be the standard first-line option [15,19].

There are several studies and case reports describing surgical approaches for resecting desmoid tumours when present in the abdominal wall but the best surgical strategy is controversial and it is dependent on the patient's background and the surgeon's preference [2,3,13,17]. In our case the patient was young, with an active life and she already had a baby; the mass was very painful so we decided, after a consult with our oncologist, for the surgical strategy.

## 4 Conclusion

The optimal treatment strategy for desmoid tumours still remains unclear. Surgery is the primary treatment and a radical resection with free margins remains the principal determinant of outcome [12,15,19]. Non-surgical approaches may be useful for adjuvant therapy in patients with unresectable lesions [14,20]. Further studies are needed in order to establish the best strategy, but the approach must always be multidisciplinary as we did in our case.

## Units

International System of Units (SI).

## Funding

The authors declare that no sources of funding have been requested for this research.

## Ethical approval

The authors declare that this study is exempt from ethical approval.

## Consent

Written informed consent was obtained from the patient for publication of this case report and accompanying images. A copy of the written consent is available for review by the Editor-in-Chief of this journal on request.

## Registration of research studies

The authors declare that no registration is needed for this work.

## Guarantor

Mr Bruno Scotto is the Guarantor for the work.

## Provenance and peer review

Not commissioned, externally peer-reviewed.

## CRediT authorship contribution statement

**Francesca Ascari:** Data curation, Writing - original draft. **Silvia Segattini:** Visualization, Investigation. **Michele Varoli:** Supervision. **Massimo Beghi:** Software, Validation. **Simone Muratori:** Conceptualization, Methodology, Software. **Bruno Scotto:** Writing - review & editing, Supervision. **Marco Gasperoni:** Data curation, Software, Validation.

## Declaration of Competing Interest

The authors declare that have no conflicts of interest.
